# 1.2 MV/cm pulsed electric fields promote transthyretin aggregate degradation

**DOI:** 10.1038/s41598-020-68681-0

**Published:** 2020-07-20

**Authors:** Gen Urabe, Takashi Sato, Gomaru Nakamura, Yoshihiro Kobashigawa, Hiroshi Morioka, Sunao Katsuki

**Affiliations:** 1 Graduate School of Science and Technology, Kumamoto University, Kumamoto, 860-8555 Japan; 2 Institute of Pulsed Power Science, Kumamoto University, Kumamoto, 860-8555 Japan; 3 Department of Analytical and Biophysical Chemistry, Kumamoto University, Kumamoto, 862-0973 Japan

**Keywords:** Biophysics, Molecular biophysics, Supramolecular assembly, Computational models, Computational biology and bioinformatics, Protein folding, Protein aggregation

## Abstract

Numerous theoretical studies have been conducted on the effects of high-voltage electric fields on proteins, but few have produced experimental evidence. To acquire experimental data for the amyloid disassemble theory, we exposed transthyretin aggregates to 1 ns 1.26 MV/cm pulsed electric fields (PEFs) to promote transthyretin degradation. The process produced no changes in pH, and the resulting temperature increases were < 1 °C. We conclude that the physical effects of PEFs, rather than thermal or chemical effects, facilitate aggregate degradation.

## Introduction

Biological reactions to electric and pulsed electric fields (PEFs) have been reported^1–6^. Although much discussion has focused on membrane dynamics and damage, attention is now shifting to proteins because membranes and their associated proteins, such as channels, pumps, actin cables, and microtubules, respond to electric fields, both theoretically and experimentally^7–12^. However, most previous studies analyzed cell proteins^13–15^ and did not consider the contributions of cellular activities under electric fields. For example, protein phosphorylation in PEFs may be the result of trans-membrane calcium influx, which activates calcineurin, and promotes kinase activation^16^. To examine direct electrical effects, purified proteins must be exposed to PEFs, but membrane and membrane-associated proteins may be under a field of ≥ 1 MV/cm. The potential benefits presented by analysis of proteins under electric fields stronger than 1 MV/cm have interested many researchers. However, because it is technically challenging to apply strong electric fields to liquid samples, most previous studies have used numerical calculations^17–21^. According to these calculations, proteins change their three-dimensional structures under a static electric field of 1 MV/cm. Therefore, we based our efforts on amyloid destruction theory, which speculates that amyloids are destroyed under fields stronger than 1 MV/cm that last longer than 400 ns^22^. Many biologists and biochemists are skeptical of this theory because it is difficult to prove experimentally, and amyloid collapse has yet to be demonstrated. However, verifying this theory would constitute a significant advance in biology, biochemistry, and bioelectrics. Pandey et al. presented results to support the theory, but they applied a weaker electric field of 230 kV/cm for longer than 40 h. They used electrodes covered by Teflon and polydimethylsiloxane to prevent breakdown, both of which weaken electric fields in solution^23^. In this paper, we describe a nanosecond high-voltage generator and a customized chamber with a parallel, gold coated 1-mm electrode gap. We investigated the thermal, chemical, and physical effects of PEFs by applying 1,000 pulses at 1 ns, 1.26 MV/cm to transthyretin aggregates.

## Results

### Transthyretin aggregate exposed to PEF

**Figure 1:**
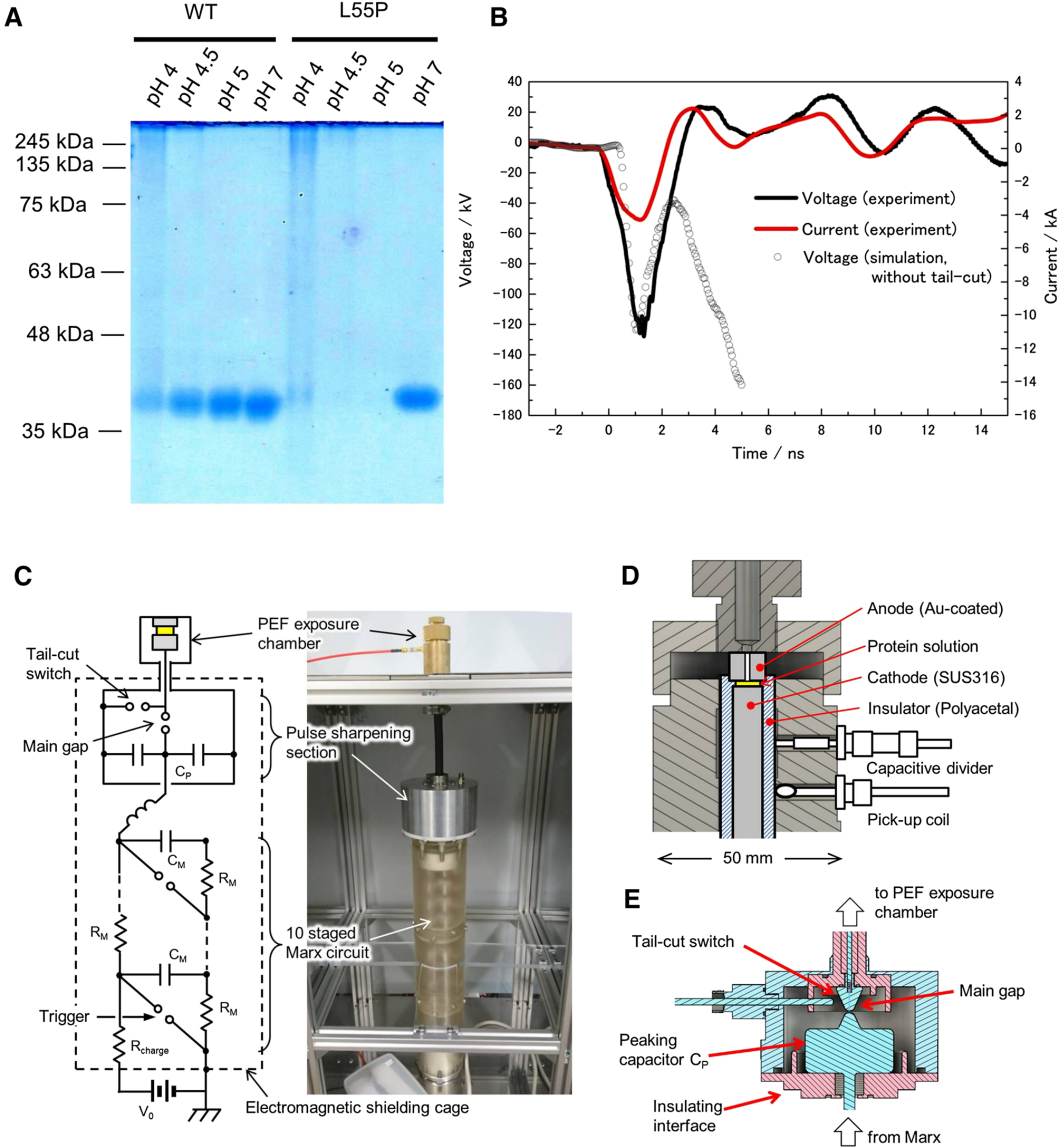
Transthyretin aggregate formation and pulse formation. (**A**) Native PAGE of transthyretin aggregates. Transthyretin of WT and L55P mutants formed aggregates with molar weights > 245 kDa at pH 4 and 37 °C for 3 days. Transthyretin concentrations were 0.2 mg/mL. The full photograph without contrast of gel electrophoresis is in SI Fig. S1A. (**B**) Voltage and current waveform. Both are the average of 20 waveforms. The maximum voltage was 126 kV, which was close to that of a simulation, and the maximum current was 4.0 kA. The voltage in the simulation rose after the main pulse because we did not set tail-cut switch in the simulation. We therefore erased the waveform after 5 ns. (**C**,**D**) Our machine combined a 10-stage Marx circuit and a pulse sharpening section. (**E**) The detailed structure of the pulse sharpening section, composed of a peaking capacitor, a gap switch, and a tail-cut switch. The insulators are red and the electric conductors are cyan. Detailed explanations of each part, the methods to acquire the signals, and the structure of the sample holder are found in SI Fig. S2. Image J adjusted (**A**).

**Figure 2:**
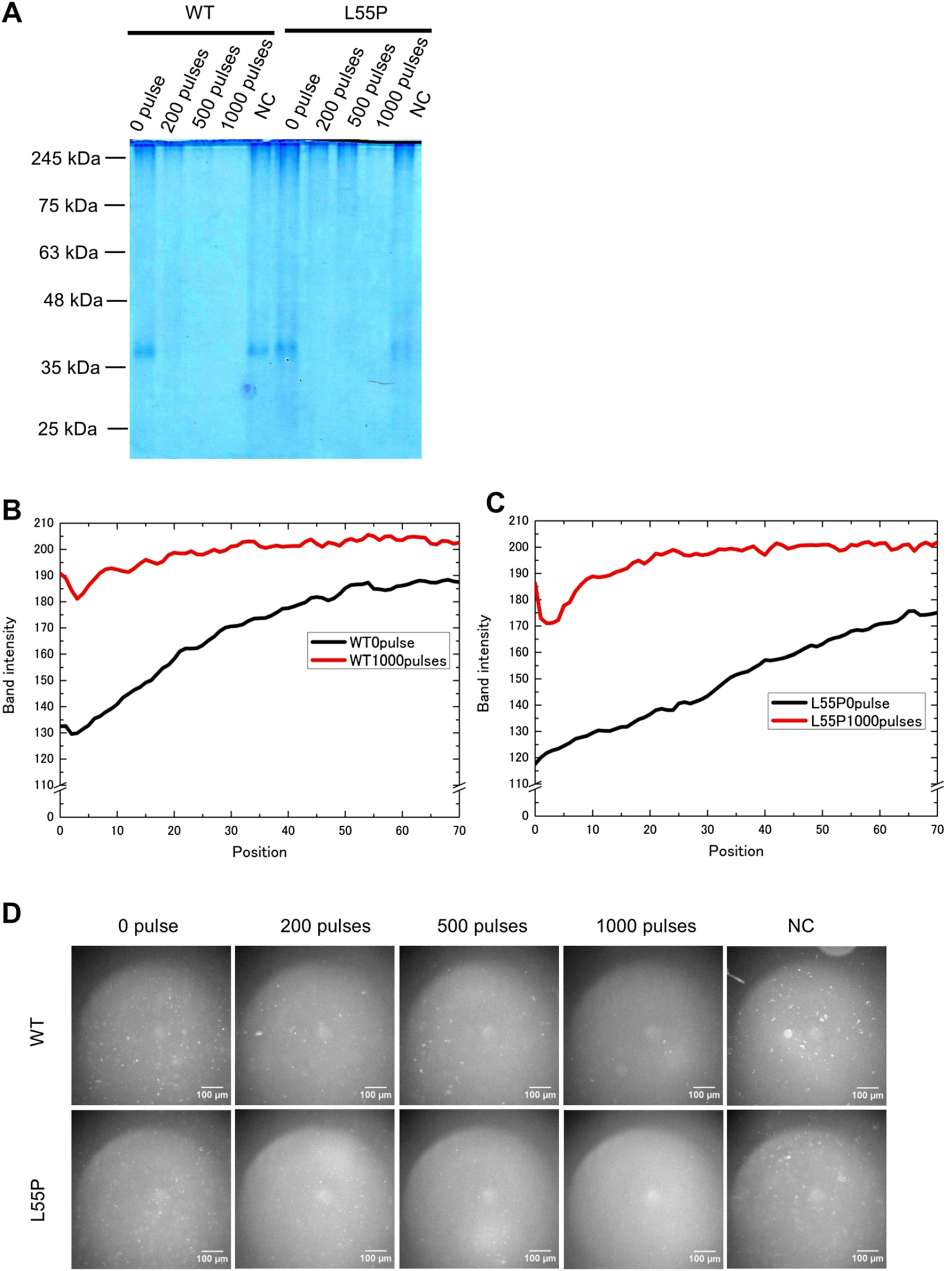
Transthyretin aggregate disappeared after exposure to 1,000 pulses at 1.26 MV/cm. (**A**) Aggregate of WT and L55P mutants disappeared after being exposed to 1,000 pulses at 1.26 MV/cm. Aggregate concentrations during PEF treatment were 0.2 mg/mL. NC was an aggregate solution (0.4 mg/mL) mixed with PEF-treated triple-diluted HEPES buffer at a volume ratio of 1:1. 1 µg of aggregate was in the native PAGE gels. Aggregate distributions of WT (**B**) and L55P mutants (**C**) of 0 pulse and 1,000 pulses at 1.26 MV/cm. The full photograph without contrast of gel electrophoresis is in SI Fig. S1B. (**D**) Aggregate fluorescent photos with a green fluorescence filter. Application of 1,000 pulses at 1.26 MV/cm reduced fluorescent dots. The white scale bar represents 100 μm. Each experiment was performed more than twice. Image J adjusted (**A**,**D**), and gained the distributions of (**B**,**C**).

Transthyretin formed aggregate proteins at a pH of 4 and 37 °C (Fig. 1A). Our generator produced a voltage waveform of 126 kV with a duration of 1 ns. A current emerged with a phase lead during voltage application in both an experiment and a simulation, suggesting the sample reacted as a resistive load with a capacitive element (Fig. 1B). The ratio of reflection voltages to main voltage was 1/10, and the power ratio was 1/48, both of which are small. We therefore assumed the main pulse had the strongest impact. Our machine combined a 10-staged Marx circuit and pulse sharpening section (Fig. 1C–E). The electrode distance was 1 mm, making the maximum electric field 1.26 MV/cm. Application of 1,000 pulses of a PEF at 1.26 MV/cm appeared to erase transthyretin aggregates in both the wild-type (WT) and L55P mutants, supporting the aggregate destruction theory (Fig. 2A–C). We also observed the disappearance of fluorescent dots after PEF treatment, likely because transthyretin aggregates bind to thioflavin-T to emit green fluorescence (Fig. 2D).

### Effects of reactive oxygen species, liquid pH, and liquid temperature can be ignored

**Figure 3:**
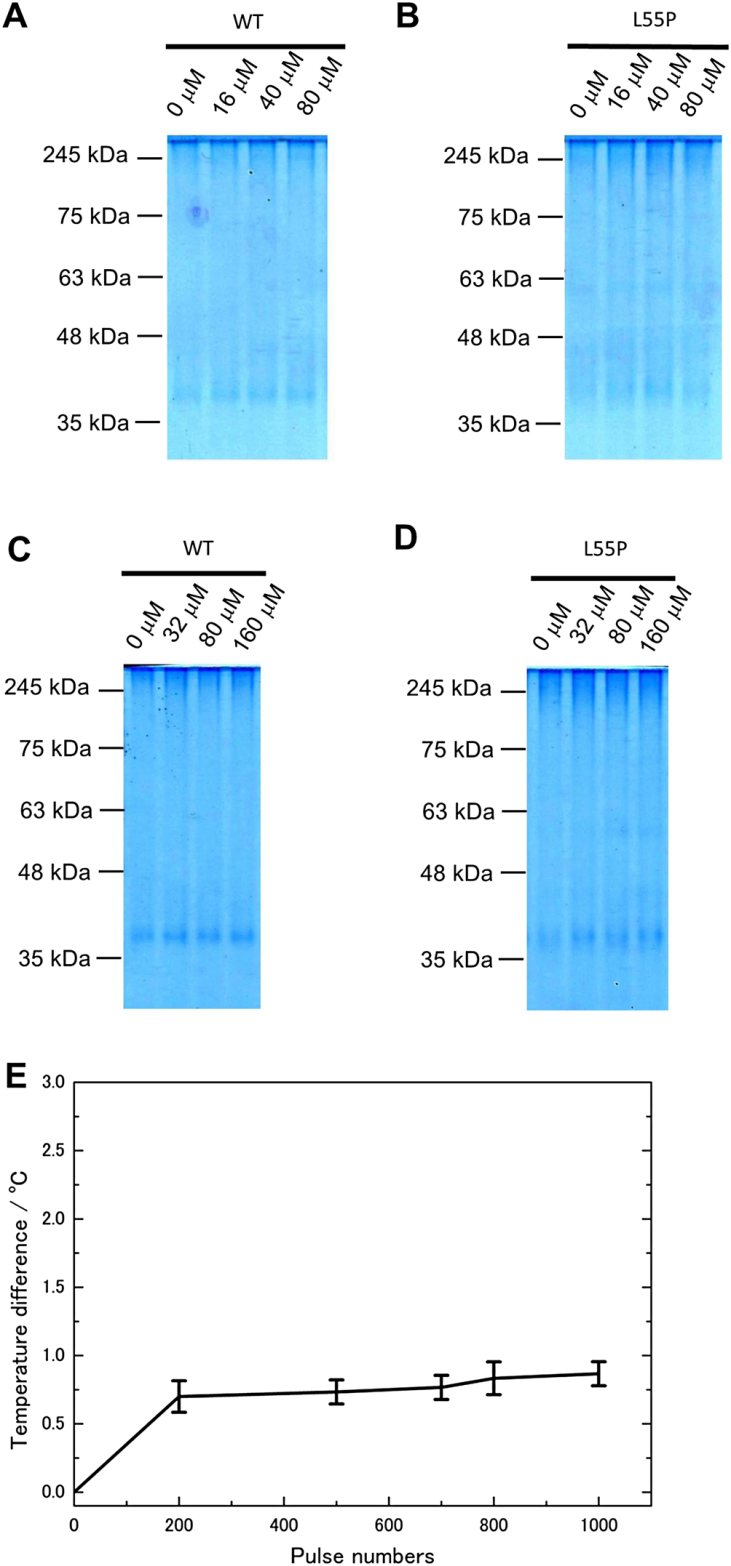
Consideration of typical chemical effects. H_2_O_2_ did not affect transthyretin aggregates on either WT (**A**,**C**) or L55P (**B**,**D**). Two pulses at 1.26 MV/cm generated 0.08 ± 0.01 μM of H_2_O_2_. Concentrations in (**A**,**B**) were pulse number times 0.08 ± 0.01 μM. Those of (**C**,**D**) were twice the values of (**A**,**B**). Each experiment was performed once, but L55P at 160 μM was performed twice. (**A**–**D**) Native PAGE with 1 μg of aggregate in the gels. All aggregate concentrations during H_2_O_2_ treatment were 0.2 mg/mL. The full photographs without contrast of gel electrophoresis are in SI Fig. S3. (**E**) Temperature rise at each pulse timing. The temperature increase at 1,000 pulses was < 1 °C. N = 3 and error bars were standard error of the mean. Details of the temperature measurements are supplied in SI Fig. S3E,F. Image J adjusted (**A**–**D**).

Because PEF generates reactive oxygen species, mainly in the form of H_2_O_2_, in liquid samples^24^, we measured the H_2_O_2_ that emerged after two pulses of 1.26 MV/cm PEFs, detecting 0.08 ± 0.01 μM H_2_O_2_ (Table 1). Assuming that the amount of H_2_O_2_ varies by pulse number, each concentration should have been less than the pulse number times 0.08 ± 0.01 μM. The aggregate did not collapse at any H_2_O_2_ concentration, even though at twice more than Fig. 3A,B (Fig. 3A–D). A comparison of the liquid pH before and after application of 1,000 pulses revealed no change from the initial value of 6.8 (Table 1). Furthermore, 1,000 pulses raised the liquid temperature by < 1 °C (Fig. 3E). This indicates that direct electrical stress, rather than chemical or thermal effects, induced aggregate destruction.

**Table 1:** The amount of hydrogen peroxide that emerged after two pulses at 1.26 MV/cm and solution pH before and after PEF treatment.

Hydrogen peroxide/μM	pH before PEF	pH after PEF
0.08 ± 0.01	6.8	6.8

All parameters are an average of N = 3. Error was standard error of the mean. All pH measurements repeatedly resulted in a value of 6.8.

### Stronger electric fields promoted aggregate disassembly

**Figure 4:**
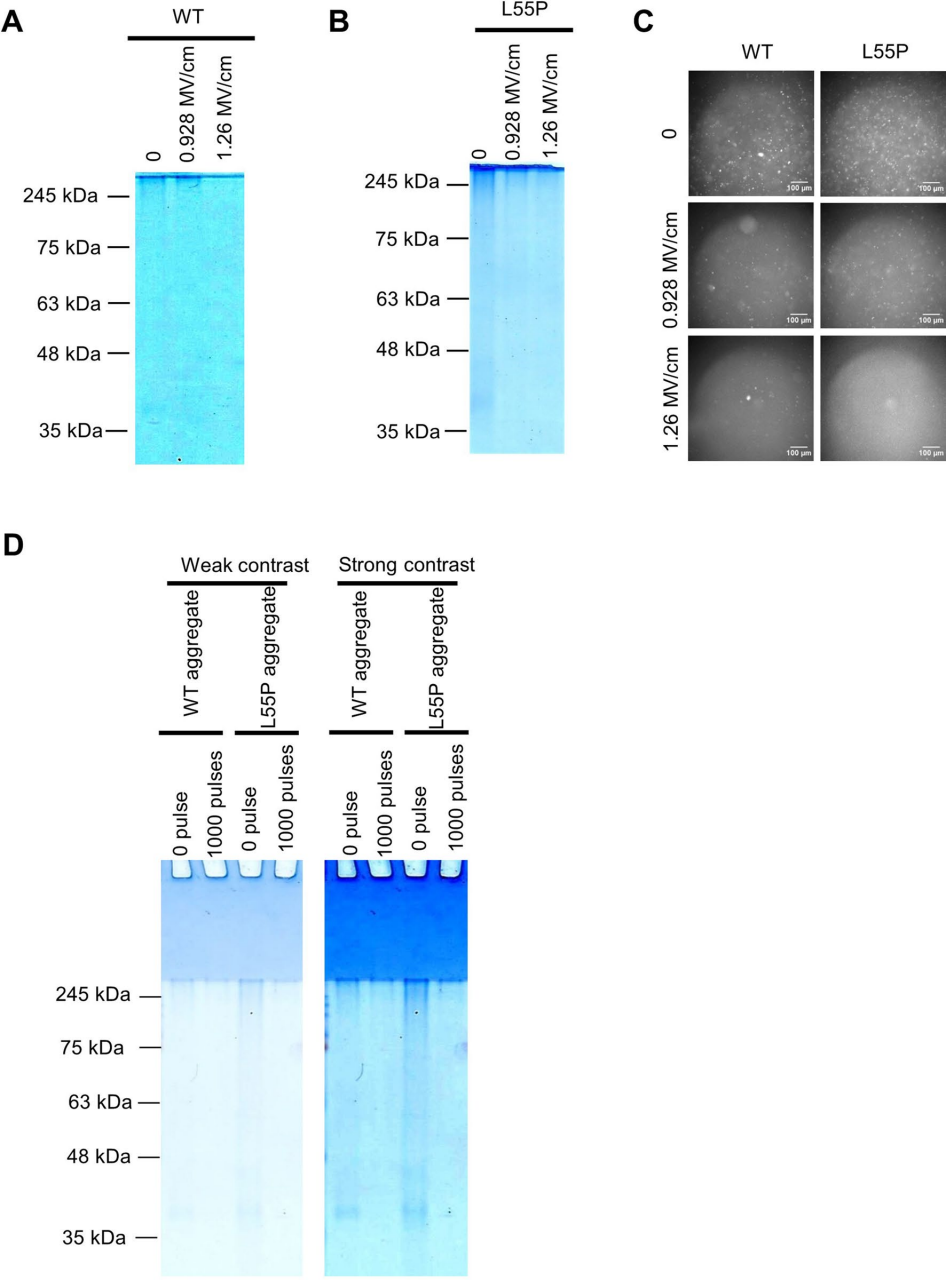
Effects of electric field strength and the possibility of PEF-promoted aggregation. A weaker electric field (0.928 MV/cm) was less effective than a 1.26-MV/cm PEF in both WT (**A**) and L55P mutants (**B**). This tendency was the same as in fluorescent analysis with a green fluorescence filter (**C**). The pulse number was 1,000 in (**A**–**C**). (**D**) If proteins aggregated into a large molecule, strong bands emerged on the well bottom of the stacking gel. Such a signal did not emerge, although the aggregate disappeared. (**A**–**C**) were performed twice but (**D**) just once. (**A**,**B**,**D**) Native PAGE with 1 μg of aggregate in the gels. The full photographs without contrast of gel electrophoresis are in SI Fig. S4. Concentrations of aggregates during PEF treatment were 0.2 mg/mL. Image J adjusted (**A**–**D**).

Exposing the aggregate to 1,000 pulses of a 1.26-MV/cm PEF destroyed it more effectively than did 0.928 MV/cm pulses in a manner consistent with the proposed theory (Fig. 4A–C). Values of the electric field of 1.26 and 0.93 MV/cm were chosen based on the characteristics of the spark-gap driven high voltage pulse generator, operation of which is generally sensitive to voltage. The higher (126 kV) and lower limits (93 kV) produced an identical and stable waveform in our present device.

An electric field has the potential to form new covalent bonds in egg whites^25–27^. A 1.26-MV/cm PEF may promote assembly of an aggregate with a molar weight too large to detect by gel electrophoresis, reducing the aggregate electrophoresis band. A stacking gel photograph showed heavy molecules did not aggregate after 1,000 pulses of 1.26 MV/cm PEF, again supporting the amyloid disassembly hypothesis (Fig. 4D).

### PEF digested aggregate-derived but not tetramer-derived transthyretin

**Figure 5:**
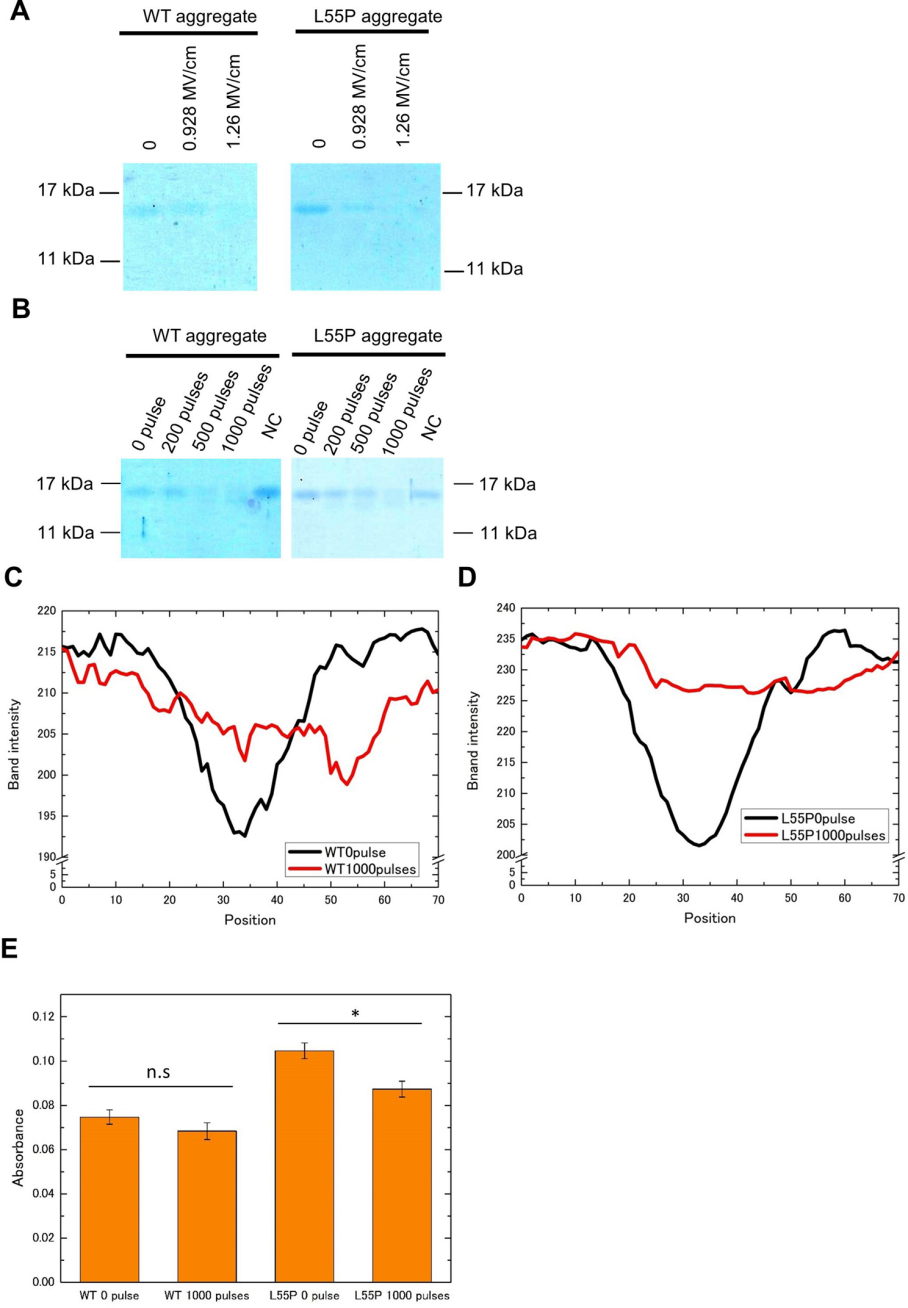
Aggregate-derived transthyretin subunit analysis with SDS-PAGE. Application of 1,000 pulses at 1.26 MV/cm destroyed aggregate-derived transthyretin subunits and weaker electric fields (**A**), whereas fewer pulses at 1.26 MV/cm decreased the ability (**B**) in both WT and L55P mutants. NC is negative control. The aggregate concentration during PEF treatment was 0.2 mg/mL in A and B. Aggregate-derived subunit band-intensity distributions of WT (**C**) and L55P mutants (**D**) of 0 pulse and 1,000 pulses at 1.26 MV/cm. 0.35 μg of aggregate in the gels. The full photographs without contrast of gel electrophoresis are in SI Fig. S5. (**E**) Comparing the absorbance of aggregates using BCA, the L55P mutant exhibited a significant decrease after 1,000 pulses at 1.26 MV/cm but WT proteins did not (n.s means no significant, *p < 0.01, N = 3). Image J adjusted (**A**,**B**), and gained the distributions of (**C**,**D**).

To examine whether transthyretin persisted after PEF application, we performed sodium dodecyl sulfate (SDS)-polyacrylamide gel electrophoresis (PAGE) in a reducing condition. The subunit band of transthyretin disappeared after 1,000 pulses of a 1.26-MV/cm PEF in both WT and L55P mutants, with a dramatic change in band-intensity distributions, indicating that aggregate transthyretin was digested (Fig. 5A–D). Measurement of absorbance using bicinchoninic acid (BCA) to detect peptide bonds revealed that the absorbance of an L55P aggregate after 1,000 pulses at 1.26 MV/cm decreased significantly, suggesting that the PEF broke peptide bonds to digest transthyretin. The WT protein was not associated with significant reductions, but treated samples tended to reduce signal strength (Fig. 5E).

**Figure 6:**
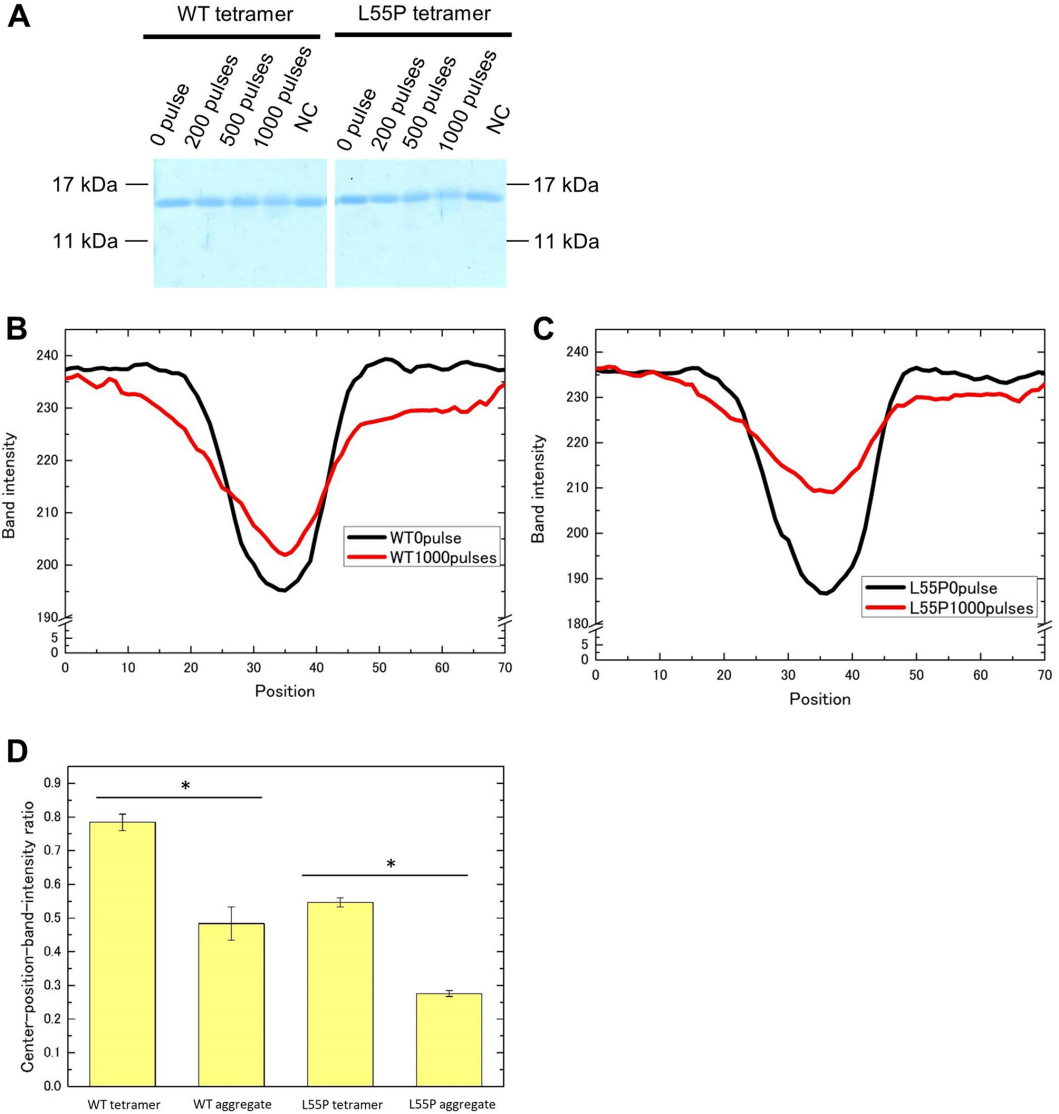
Tetramer-derived transthyretin subunit analysis with SDS-PAGE. (**A**) 1,000 pulses at 1.26 MV/cm slightly reduced the tetramer-derived subunit band. NC is negative control. Tetramer-derived subunit band-intensity distributions of WT (**B**) and L55P mutants (**C**) of 0 pulse and 1,000 pulses at 1.26 MV/cm. The tetramer concentration during PEF treatment was 0.1 mg/mL in A. The analysis in (**A**) was performed twice with SDS-PAGE. A 0.35-μg sample of transthyretin was in the gels. The full photographs without contrast of gel electrophoresis are in SI Fig. S6. (**D**) Center-position intensity ratios of tetramer- or aggregate-derived subunits of WT and L55P mutants (*p < 0.01, N = 10). Details for calculating the ratios are supplied in SI Fig. S6C. Image J adjusted (**A**), and gained the distributions of (**B**,**C**).

Normal tetramer transthyretin exhibited reduced affects compared with aggregate transthyretin; the tetramer transthyretin subunit band disappeared at 1,000 pulses of a 1.26-MV/cm PEF in both WT and L55P, with small changes in band-intensity distributions (Fig. 6A–C). To evaluate the degrees of decrease in subunit band intensities, we calculated center-position intensity ratios (SI Fig. S6C). The results indicate that the aggregate ratios were less than those of the tetramers (Fig. 6D).

Although non-specific bands between 63 and 75 kDa usually appear in reduced SDS-PAGE, signal intensity and subunit band disappearance exhibited no apparent correlation (SI Figs. S5C,D; S6A,B).

## Discussion

### Amyloid destruction theory requires improvement

**Figure 7:**
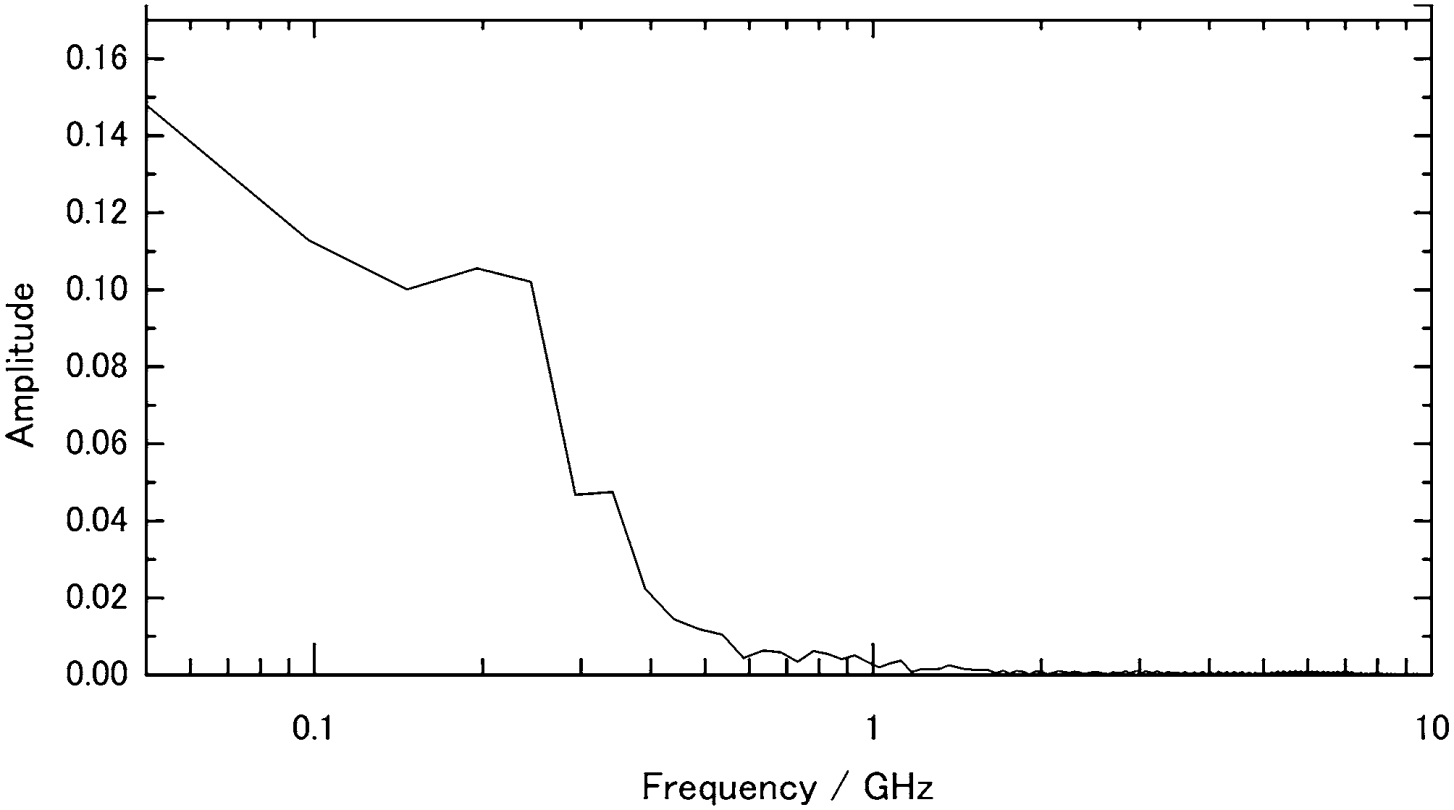
Fourier transformation of voltage in Fig. 1B. Frequency distribution from 0.05 to 10 GHz.

Our results not only support the amyloid destruction theory but also suggest the theory should be modified to explain protein digestion, which had not been predicted and did not occur in tetramer transthyretin. The differences between aggregate and tetramer transthyretin in SDS-PAGE can be explained by examining the charge density derived from charged and polarized amino acids and the mechanical oscillation frequency. The amyloid structure appeared to be much denser than the tetramer structure, implying that aggregates may be subject to greater electrical stress due to higher charge density^28,29^. In terms of oscillation frequency, supramolecular (whole-structure) vibration frequencies in microtubules and filaments cover the MHz-to-GHz range. However, molecular vibrations of normal proteins derived from weak bonds and atoms around the bonds occur in the THz range^30,31^. The PEFs' main frequencies were near 50 MHz and from 150 to 250 MHz (Fig. 7). Frequencies near 50 MHz may constitute the flat portion of the voltage waveform in Fig. 1B, such as from − 3 to 0 ns. However, 200 MHz constitutes the lower part of the main pulse, and 250 MHz is an upper element of the waveform (SI Fig. S7). It was included in the frequency zone of supramolecular vibrations in Refs.^28,29^. PEFs of 1.26 MV/cm lasting 1 ns may efficiently promote sympathetic aggregate vibrations and, consequently, induce collapse of covalent transthyretin bonds. To examine this hypothesis in future experiments, we anticipate measuring impedance frequency characteristics of tetramer and aggregate transthyretin, adjusting the PEF duration to longer than 2 ns.

One of the possible reasons the WT aggregate was not significantly reduced with BCA (Fig. 5E) is that the amount of WT aggregate was less than that of L55P (Figs. 1A, 4D), and the amount of WT decrease was approximately 50%, which was too small to constitute a significant decrease (Fig. 6D).

### Local pH shift on electrode surface vicinity

Non-buffer solutions can cause pH values to jump on a cathode surface and plunge on the anode side, whereas a buffer solution produces smaller effects^32–34^. Transthyretin aggregate formed at pH 4 (Fig. 1A), aggregates at pH 9 remained (Figs. 2D, 4C), and aggregate was in a HEPES buffer during PEF exposure. These results suggest that a shift in pH may not affect aggregate degradation, regardless of whether a local pH shift occurs.

### Small thermal effects due to heat release to the electrodes

The maximum voltage and current were 126 kV and 4.0 kA, respectively. The specific heat of water is 4.2 J/gK, and the sample was a cylinder with a diameter of 5.5 mm and a height of 1 mm. The theoretical temperature jump is therefore 5.1 °C, but it will heat the sample only to 23 °C after one pulse because the room temperature was 18 °C. This thermal effect will not affect aggregate digestion because transthyretin aggregates form at 37 °C, and they persist at 0 °C. Furthermore, the pulse exposure frequency was 1.7 Hz, making it relatively easy to release heat to the electrodes. Figure 3E depicts a temperature shift of < 1 °C even after 1,000 pulses.

### Effect of electrical double layer (EDL) on electric field in the solution

It is possible for a voltage drop to occur at electrical double layers (EDL) and at liquid-electrode interfaces^35^. Anions and cations in a conductive solution are accumulated in the vicinity of the anode and cathode surfaces, respectively, under an external electric field, and form EDLs on both anode and cathode surfaces. The EDLs are regarded as capacitors and have a large potential gradient in accordance with the development of charge accumulation. Since the charge accumulates in the microsecond timeframe, the charge accumulation is not developed significantly under an external field with the frequency exceeding 1 MHz^36–38^. Therefore, the measured voltage in this study can be reasonably applied to the protein solution.

## Conclusions

This paper not only supports the amyloid disassemble theory that electric fields stronger than 1 MV/cm promote aggregate collapse but suggests a need to add a protein digestion hypothesis to the theory. Our experiment confirmed that aggregate degradation was caused by the physical effects of PEF, not the chemical or thermal effects.

## Materials and methods

### Transthyretin preparation and aggregate formation

Recombinant WT and L55P TTR were expressed and purified from *Escherichia* *coli* BL21 (DE3) as described previously^39^. A 0.4-mg/mL sample of transthyretin in a HEPES buffer (50 mM HEPES, 150 mM NaCl, pH 6.8) was mixed with an acetate buffer (200 mM sodium acetate, 50 mM NaCl, pH 4) at a ratio of 20:20 μL and kept at 37 °C for 3 days. The pH 4.5 and pH 5 acetate buffers had the same composition, but a pH 7 solution was composed of a 50-mM NaCl solution. A 40-µL sample of aggregate was centrifuged at 20,000*g* at 4 °C for 5 min. After removing the supernatants, sediment aggregates were dissolved in a triple-diluted HEPES buffer (17 mM HEPES, 50 mM NaCl, pH 6.8) for pulse application.

### 1.26 MV/cm PEF generation

We used a nanosecond pulse to prevent plasma formation in the PEF exposure chamber. Our high-voltage nanosecond pulse generator is similar in structure to the one described in the reference^40^ and consists of a spark gap-driven 10-stage Marx circuit with an output capacitance of 94 pF; and a pulse-sharpening section, including a coaxial capacitor with a capacitance of 12 pF, a spark gap as the output switch, and a tail-cut switch to reduce the pulse to 1 ns. The Marx circuit was placed in a polycarbonate container pressurized to 0.57 MPa with nitrogen gas and charged to 15 kV using a high-voltage DC supply (HAR-50R0.6, Matsusada). The pulse-peaking capacitor and the tail-cut switch were placed in an aluminum container pressurized to 0.5 MPa with sulfur hexafluoride gas. The elevated high-voltage pulse was delivered to the pulse-peaking capacitor to be doubled and then quickly released to a 50-Ω coaxial cable leading to the PEF exposure chamber by closing the output switch (Fig. 1C–E; SI Fig. S2A–E).

Voltage and current sensors used a capacitive divider and a pick-up coil, both of which were integrated in the exposure chamber, respectively. The signals were acquired by a 16-GHz oscilloscope (DPO71604C, Tektronix). The voltage sensor was calibrated by a calibrated resistive divider with a range of 50 kV. Numerical simulation of the pulse delivery using CST Studio Suite (SIMULIA) validated the measurements (SI Fig. S2F–O).

The 1 ns, 126 kV pulses were delivered repeatedly to the coaxial PEF exposure chamber with a 1-mm gap, and parallel-plane electrodes 5.5 mm in diameter, to generate an electric field exceeding 1 MV/cm (SI Fig. S2P–R). Deviations of maximum voltage amplitude during the repetitive operation were approximately 5%. The electrodes were made of stainless steel (SUS316), and the anodic electrode was gold coated to minimize the chemical influence of metal ions from the electrodes. The electrical resistance and the estimated capacitance of the electrodes, including samples, were 77 Ω and 17 pF, respectively, resulting in a total impedance of 34 Ω at 250 MHz. The measured voltage at the electrodes was 126 kV, which was 20% lower than the expected voltage at a load of 50 Ω, because of negative mismatching.

The pulse repetition rate was fixed at 1.7 Hz to prevent a significant temperature rise during PEF exposure. Negative controls (NCs) were aggregate or tetramer transthyretins dissolved in a 1.26 MV/cm nsPEF-treated triple-diluted HEPES buffer (17 mM HEPES, 50 mM NaCl, pH 6.8). The conductivity of the solution was 0.578 S/m (LAQUAtwin-EC-33, HORIBA).

### H_2_O_2_ detection, pH measurement, and temperature measurement

We used an Amplite Fluorimetric Hydrogen Peroxide Assay Kit *Near Infrared Fluorescence* from CosmoBio to measure H_2_O_2_ concentrations. We prepared a working solution (Amplite IR Peroxide Substrate, 0.8 U/mL peroxidase) and mixed the solution with miliQ at a ratio of 100:100 μL. The mixed solution was exposed to 1.26 MV/cm for two pulses and collected. Because the maximum treatable amount was 25 μL, we combined nine samples of two-pulse solutions to prepare 200 μL of the treated sample. A Quantus fluorometer measured the sample fluorescence with a red fluorescence filter to calculate the concentration of H_2_O_2_.

A LAQUAtwin compact pH meter was used to measure the pH of pre- and post-treated liquids. Because at least 100 μL is required for a measurement, we prepared five samples of 1,000-pulse solutions for the 100 μL treated samples.

An AMOTH FL-2400 fiberoptic thermometer with a FS300-2M probe measured on-time temperature of the pulse-treated solution.

The starting temperature was a room temperature of approximately 18 °C. The head of the FS300-2 M fiber probe received a fluorescent coating with a signal intensity that depended on temperature. An AMOTH FL-2400 fiberoptic thermometer exposed the coating to a laser and detected the signal intensity. As shown in SI Fig. S3E,F, the fiber probe was placed in the sample solution (triple-diluted HEPES buffer without transthyretin) through the electrode hole, and we were able to measure the temperature in real time.

### Native PAGE

Native PAGE used a 4% stacking gel (4 w/v% acrylamide/bis mixed solution 29:1, 0.125 M Tris–Cl pH 6.8, 0.09 w/v% ammonium peroxodisulfate solution, 0.08 v/v% *N*,*N*,*N*′,*N*′-tetramethylethylenediamine) and 14% separation gel (14 w/v% acrylamide/bis mixed solution 29:1, 0.375 M Tris–Cl pH 8.8, 0.09 w/v% ammonium peroxodisulfate solution, 0.08 v/v% *N*,*N*,*N*′,*N*′-tetramethylethylenediamine). Next, 5.5 µL of transthyretin solution and 5.5 µL of the sample buffer (0.1 M Tris–Cl pH 6.8, 20 v/v% glycerol, 0.025 w/v% bromophenol blue [BPB]) were mixed, and 10 µL of mixed solutions were applied to the wells of the gel. A Mini300 electric power source obtained from AS ONE applied a constant 15 mA current for 200 min for electrophoresis.

### Sodium dodecyl sulfate–polyacrylamide gel electrophoresis

SDS-PAGE used 4% stacking gel (4 w/v% acrylamide/bis mixed solution 29:1, 0.125 M Tris–Cl pH 6.8, 0.1 w/v% SDS, 0.09 w/v% ammonium peroxodisulfate solution, 0.08 v/v% *N*,*N*,*N*′,*N*′-tetramethylethylenediamine) and 15% separation gel (15 w/v% acrylamide/bis mixed solution 29:1, 0.375 M Tris–Cl pH 8.8, 0.1 w/v% SDS, 0.09 w/v% ammonium peroxodisulfate solution, 0.08 v/v% *N*,*N*,*N*′,*N*′-tetramethylethylenediamine). Transthyretin solution and a sample buffer (0.1 M Tris–Cl pH 6.8, 20 v/v% glycerol, 4 w/v% SDS, 12 v/v% 2-mercaptoethanol, 0.025 w/v% BPB) were mixed at ratio of 1:1 and heated at 100 °C for 10 min. After cooling, the samples were centrifuged at 20,000*g* for 1 min. Next, 3.5 µL of mixed solutions at 0.1 mg/mL and 7 µL of mixed solutions at 0.05 mg/mL were applied to wells of the gel. A Mini300 electric power source obtained from AS ONE applied a constant 18 mA current for 110 min for electrophoresis.

### Band-intensity distribution analysis

ImageJ software was used to analyze the electrophoresis band-intensity distributions. For the native PAGE and SDS-PAGE results, 10 vertical lines were drawn across each band, with ImageJ determining the intensity distribution through the lines for each band and presenting the average distributions measured through 10 vertical lines for each band.

### Aggregate fluorescence analysis

We used a fluorescent microscope (Leica, DMi8) combined with a digital camera (Canon, EOS 8000D) to observe aggregate fluorescence. Aggregate (0.1 mg/mL) was dissolved in thioflavin-T-glycine buffer (25 mM glycine, 10 µM thioflavin-T) and incubated on ice for 3 min without light. A green fluorescence protein filter was used.

### BCA method

A protein assay BCA kit from Wako was used to measure the amount of transthyretin aggregate. Color reaction occurred at 37 °C for 60 min. An iMark microplate reader from BIO-RAD measured absorbance at 570 nm. Aggregate absorbance, without PEF application, was calculated by subtracting the raw absorbance of a 0-pulse aggregate from that of a no-pulse triple-diluted HEPES buffer. Absorbance of 1,000 pulses at 1.26 MV/cm was acquired by subtracting the raw absorbance of treated aggregate from that of an exposed triple-diluted HEPES buffer.

### Statistical analysis

Data were presented as the mean ± standard error for n samples (as shown in Figs. 3E, 5E, 6D). Statistical analyses were performed using a two-tailed t test; p < 0.01 was considered statistically significant.

## Supplementary information

Supplementary file1 (PDF 1766 kb)

## Data Availability

All data generated or analyzed during this study are included in this published article and its Supplementary Information files.
