# Author Correction: 1.2 MV/cm pulsed electric fields promote transthyretin aggregate degradation

**DOI:** 10.1038/s41598-020-72365-0

**Published:** 2020-10-14

**Authors:** Gen Urabe, Takashi Sato, Gomaru Nakamura, Yoshihiro Kobashigawa, Hiroshi Morioka, Sunao Katsuki

**Affiliations:** 1 Graduate School of Science and Technology, Kumamoto University, Kumamoto, 860-8555 Japan; 2 Institute of Pulsed Power Science, Kumamoto University, Kumamoto, 860-8555 Japan; 3 Department of Analytical and Biophysical Chemistry, Kumamoto University, Kumamoto, 862-0973 Japan

Correction to: *Scientific Reports* 10.1038/s41598-020-68681-0, published online 20 July 2020

The original version of this Article contained errors.

In the Abstract,

“To acquire experimental data for the amyloid disassemble theory, we exposed transthyretin aggregates to 1,000 ns 1.26 MV/cm pulsed electric fields (PEFs) to promote transthyretin degradation.”

now reads:

“To acquire experimental data for the amyloid disassemble theory, we exposed transthyretin aggregates to 1 ns 1.26 MV/cm pulsed electric fields (PEFs) to promote transthyretin degradation.”

In the Introduction,

“We investigated the thermal, chemical, and physical effects of PEFs by applying 1,000 pulses at 1.26 MV/cm to transthyretin aggregates.”

now reads:

“We investigated the thermal, chemical, and physical effects of PEFs by applying 1,000 pulses at 1 ns, 1.26 MV/cm to transthyretin aggregates.”

Finally, the labels in Figures 4D, 5A, and 5B were incorrectly given as “WT amyloid” and “L55P amyloid”, and now read “WT aggregate” and “L55P aggregate” respectively.

These errors have now been corrected in the PDF and HTML versions of the Article.

