# Supplementary material for: 1.2 MV/cm pulsed electric fields promote transthyretin aggregate degradation
Source: Sci Rep. 2020 Jul 20;10:12003. doi: 10.1038/s41598-020-68681-0 (PMC7371718; doi:10.1038/s41598-020-68681-0)
Supplement: Supplementary file 1 — Supplementary file1 (PDF 1766 kb) [file 41598_2020_68681_MOESM1_ESM.pdf]

Supplementary Information for:

1.2 MV/cm Pulsed Electric Fields Promote Transthyretin Aggregate Degradation

Gen Urabe,*[1] Takashi Sato,[3] Gomaru Nakamura,[1] Yoshihiro Kobashigawa,[3] Hiroshi Morioka,[3] Sunao Katsuki*[2]

[1] Graduate School of Science and Technology, Kumamoto University, Kumamoto 860-8555, Japan

[2] Institute of Pulsed Power Science, Kumamoto University, Kumamoto 860-8555, Japan

[3] Department of Analytical and Biophysical Chemistry, Kumamoto University, Kumamoto 862-0973, Japan

*** Corresponding author**

Email address: g.urabe@st.cs.kumamoto-u.ac.jp

Email address: katsuki@cs.kumamoto-u.ac.jp

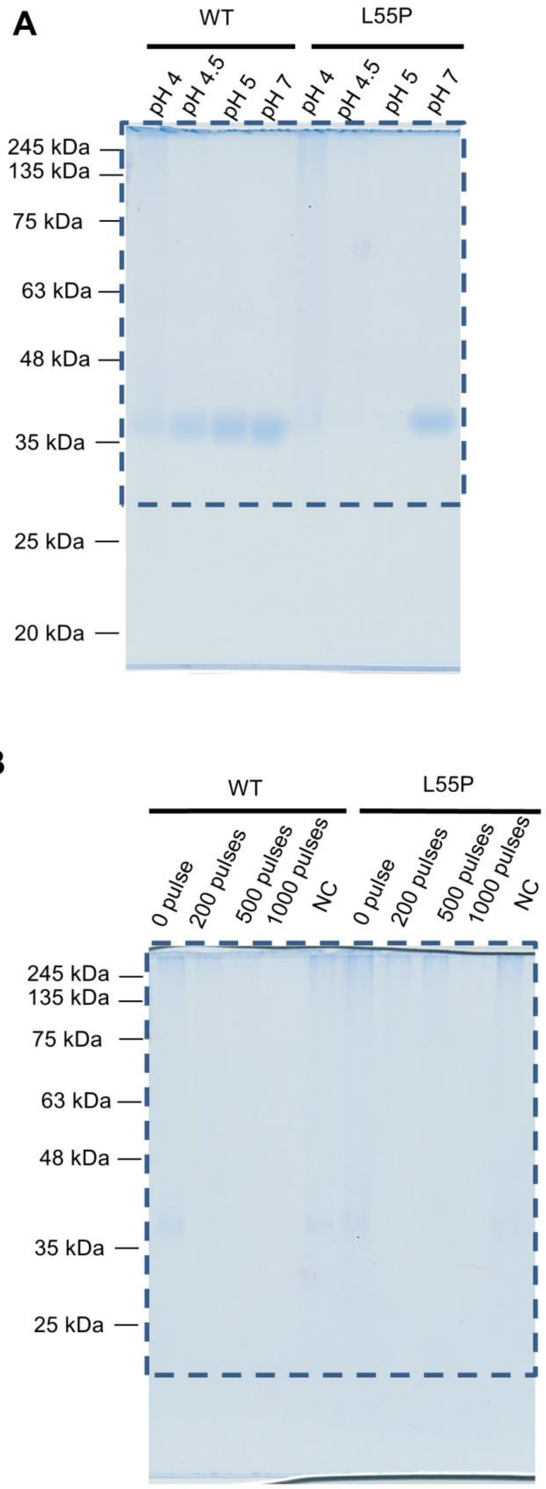

Supplementary Figure S1. Full photographs without contrast of gel electrophoresis in Figs. 1A and 2A. A: Full photograph without contrast of aggregate formation of Fig. 1A.

B: Full photograph without contrast of Fig. 2A. Image J adjusted Supplementary Figure S1A, and B.

Online Supplementary Fig. S2 depicts the generator and the acquisition of a voltage and current provided to the biochemical samples. It shows the configuration of the nanosecond high-voltage pulse generator, consisting of a 10-stage Marx circuit, peaking capacitor, and tail-cut switch, and voltage waveforms in each component. The Marx circuit generates an elevated voltage, which decays in the microsecond range (Supplementary Fig. S2C–E). The pulse-peaking capacitor compresses the pulse duration into the nanosecond range (Supplementary Fig. S2B, D, and E), and the tail-cut switch shortens the pulse and terminates the residual energy from the Marx circuit (Supplementary Fig. S2A, D, and E).

Resistive dividers cannot measure a voltage of 126 kV for longer than 1 ns because the resistance cannot divide the voltage accurately because of their floating capacitances, which are obvious under high-frequency electric fields. (Supplementary Fig. S2F–I). The voltage was divided by two capacitors in series made of polyacetal and kapton sheets, and the voltage on the kapton sheet was detected by an oscilloscope via a 1.6-kΩ resistance and two 20-dB attenuators (Supplementary Fig. S2F–I). The capacitive

divider operated under the conditions in Equation S1.

$$\frac{1}{\omega C_2} = 4.0\ \Omega \ll 1.6\ \mathrm{k\Omega}\ (\because\ \omega = 2\pi \cdot 250\ \mathrm{MHz}, C_2 = 160\ \mathrm{pF}) \tag{S1}$$

The current was measured with pick-up coil (Supplementary Fig. S2F, G, and J). Supplementary Fig. S2J specifies the following conditions:

$$V_2 = -ZI_2 = M_{12}\frac{dI_1}{dt} + L_2\frac{dI_2}{dt} \tag{S2}$$

$$M_{12}\frac{dI_1}{dt} = -ZI_2 - L_2\frac{dI_2}{dt} \tag{S3}$$

$$M_{12}\frac{dI_1}{dt} = -ZI_2$$

$$(\because\ \omega L_2 = 2.5\ \Omega \ll\ Z = 50\ \Omega, \omega \leq 2\pi \cdot 250\ \mathrm{MHz}, L_2 = 10\ \mathrm{nH}) \tag{S4}$$

$$M_{12}I_1 = -\int ZI_2 dt + \mathrm{C} \tag{S5}$$

$$I_1 = \frac{1}{M_{12}}\int V_2 dt + \mathrm{C}\quad (\because V_2 = -ZI_2) \tag{S6}$$

$$I_1 = \frac{10}{M_{12}}\int V_{osc} dt + \mathrm{C} \tag{S7}$$

The coefficient 10 in Equation (S7) is derived from the 20 dB attenuator, and the current on the sample (I_1) is an integral of the voltage (V_{osc}) shown on a Tektronix DPO71604C digital phosphor oscilloscope. Voltage was assumed with Equation (S8).

$$V_{sample} = 100000 \times \frac{536}{328} \times V_{osc} \tag{S8}$$

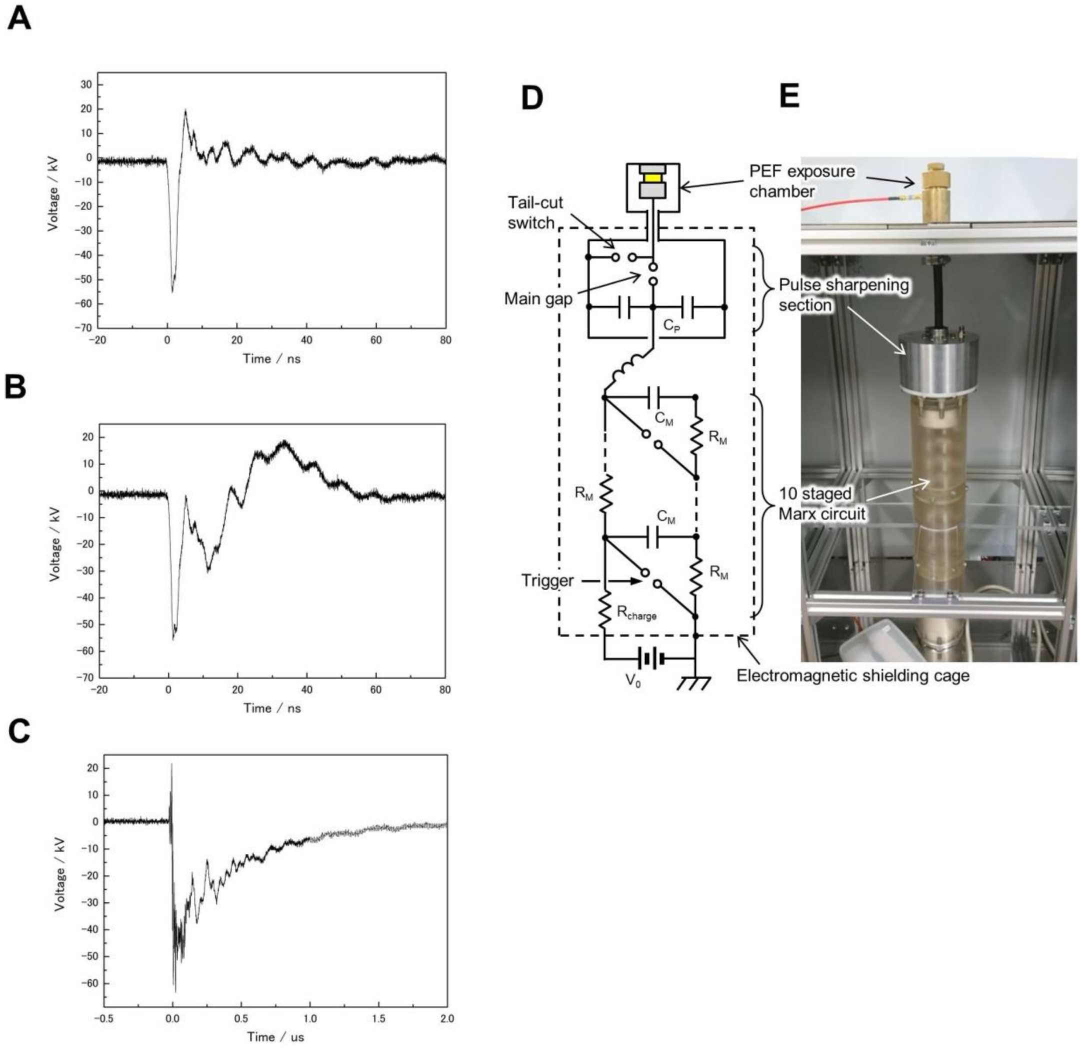

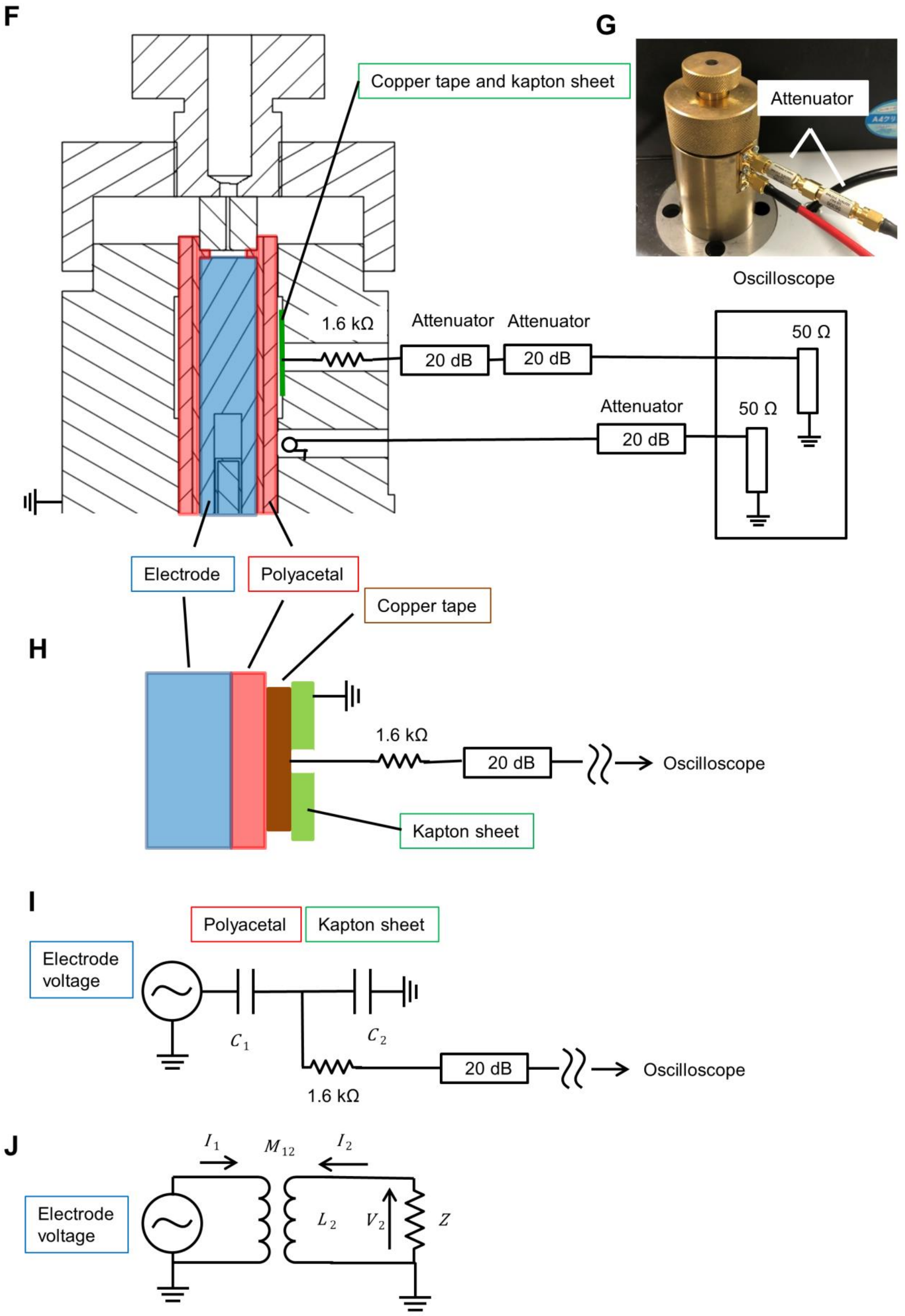

F
Copper tape and kapton sheet
1.6 kΩ
Attenuator
Attenuator
20 dB
20 dB
Attenuator
20 dB
50 Ω
50 Ω
Electrode
Polyacetal
G
Attenuator
Oscilloscope
H
Copper tape
1.6 kΩ
20 dB
Oscilloscope
Kapton sheet
I
Polyacetal
Kapton sheet
Electrode voltage
C_1
C_2
1.6 kΩ
20 dB
Oscilloscope
J
Electrode voltage
I_1
M_{12}
I_2
L_2
V_2
Z

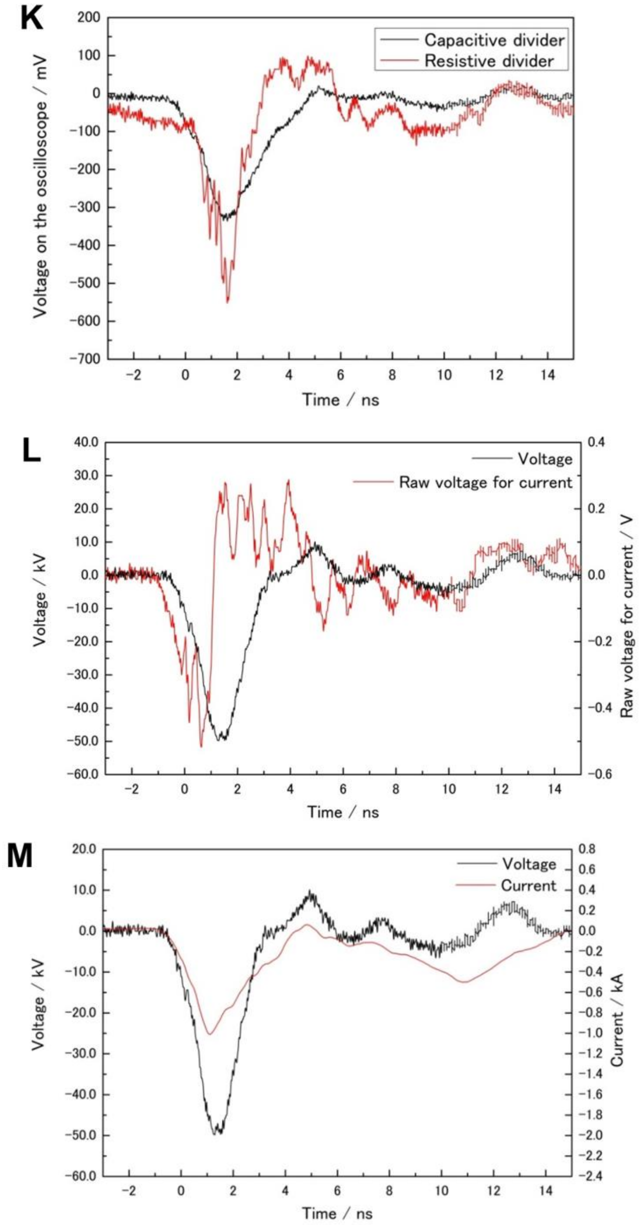
K
Capacitive divider
Resistive divider
Voltage on the oscilloscope / mV
Time / ns
L
Voltage
Raw voltage for current
Voltage / kV
Raw voltage for current / V
Time / ns
M
Voltage
Current
Voltage / kV
Current / kA
Time / ns

N

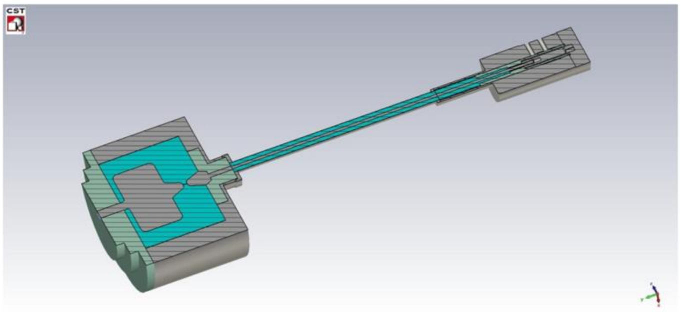

O

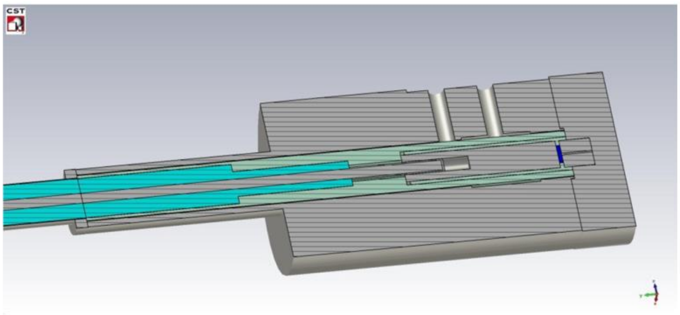

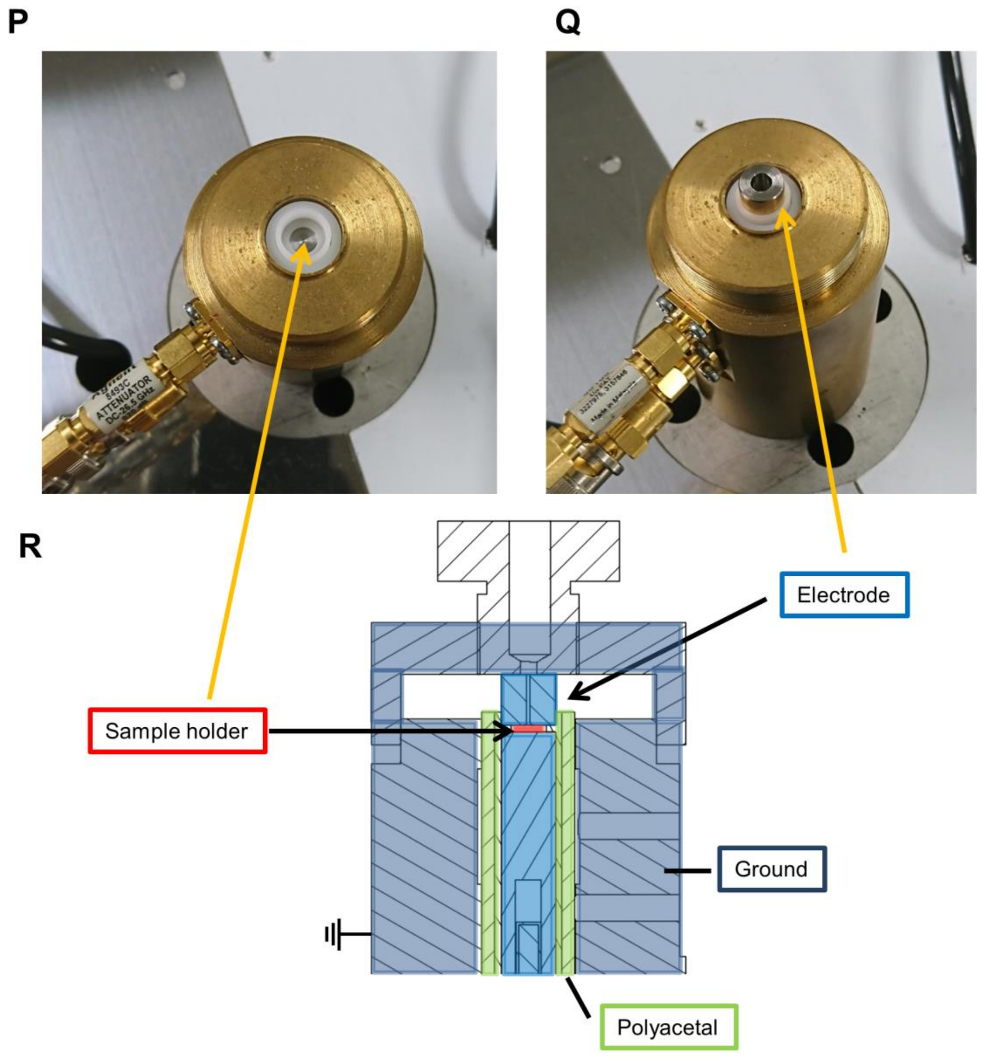

Supplementary Figure S2. The overall setup of the generator and acquisition of the signal provided to the biochemical sample. The Marx circuit generates an elevated voltage, which decays in the microsecond range (**C**). The pulse-peaking capacitor compresses the pulse duration into the nanosecond range (**B**), and the tail-cut switch shortens the pulse and terminates the residual energy from the Marx circuit (**A**). **D** is the circuit diagram, and **E** is the generator. The overall structure (**F**) and a photograph (**G**) of the acquisition of a voltage and current on the sample. The structure (**H**) and the

circuit diagram (**I**) of the capacitive divider to detect the voltage. **J**: A circuit diagram of the pick-up coil to measure the current. C_1 : the capacitance of the polyacetal capacitor, C_2 : the capacitance of the kapton capacitor. I_1 : the current in the sample, I_2 : the current on the pick-up coil, M_{12} : mutual inductance between I_1 and I_2, L_1 : self-inductance of pick-up coil, Z : impedance of the oscilloscope, and V_2: voltage on Z. **K**: Calibrating the capacitive divider by connecting a 50-Ω resistance to the electrode. When the 50 Ω resistance is exposed to 50 kV, the oscilloscope exhibits 536 mV with a resistive divider. The capacitive divider shows 328 mV. We estimated the voltage on the sample with Equation (S8). **L**: A differentiated current signal (red line) on the 50 Ω resistance under 50 kV. **M**: The current signal on the 50 Ω resistance under 50 kV was 1 kA, integrating the signal in Supplementary Fig. S2L (red line). A simulation model of the pulse sharpening section (**N**) and the sample chamber (**O**). Two cylinder electrodes face each other to form a 1-mm sample-holding space, which is embedded in a coaxial cable (**P**–**R**).

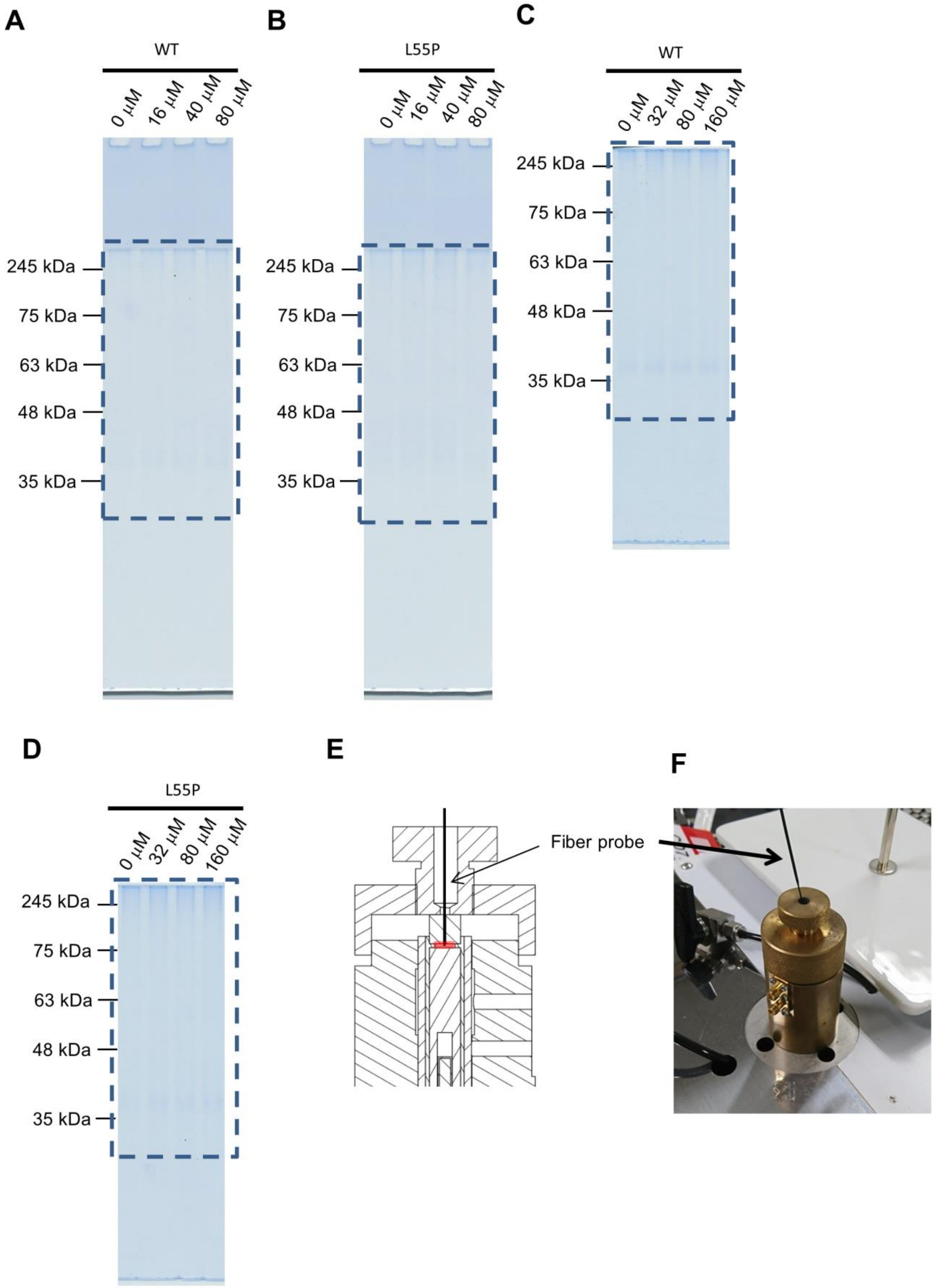

Supplementary Figure S3. Full photographs without contrast of gel electrophoresis in Fig. 3. Characters for tags are the same as those in Fig. 3 (A–D). **E** and **F**: The starting

temperature was a room temperature of approximately 18 °C. The head of an FS300-2M fiber probe coated with fluorescent coating. Signal intensity depends on temperature. An AMOTH FL-2400 fiberoptic thermometer exposes the coating to laser and detects the signal intensity. The fiber probe was placed in the sample solution (triple-diluted HEPES buffer without transthyretin) through the electrode hole, allowing for measurement of temperature in real time. Image J adjusted Supplementary Figure S3A-D.

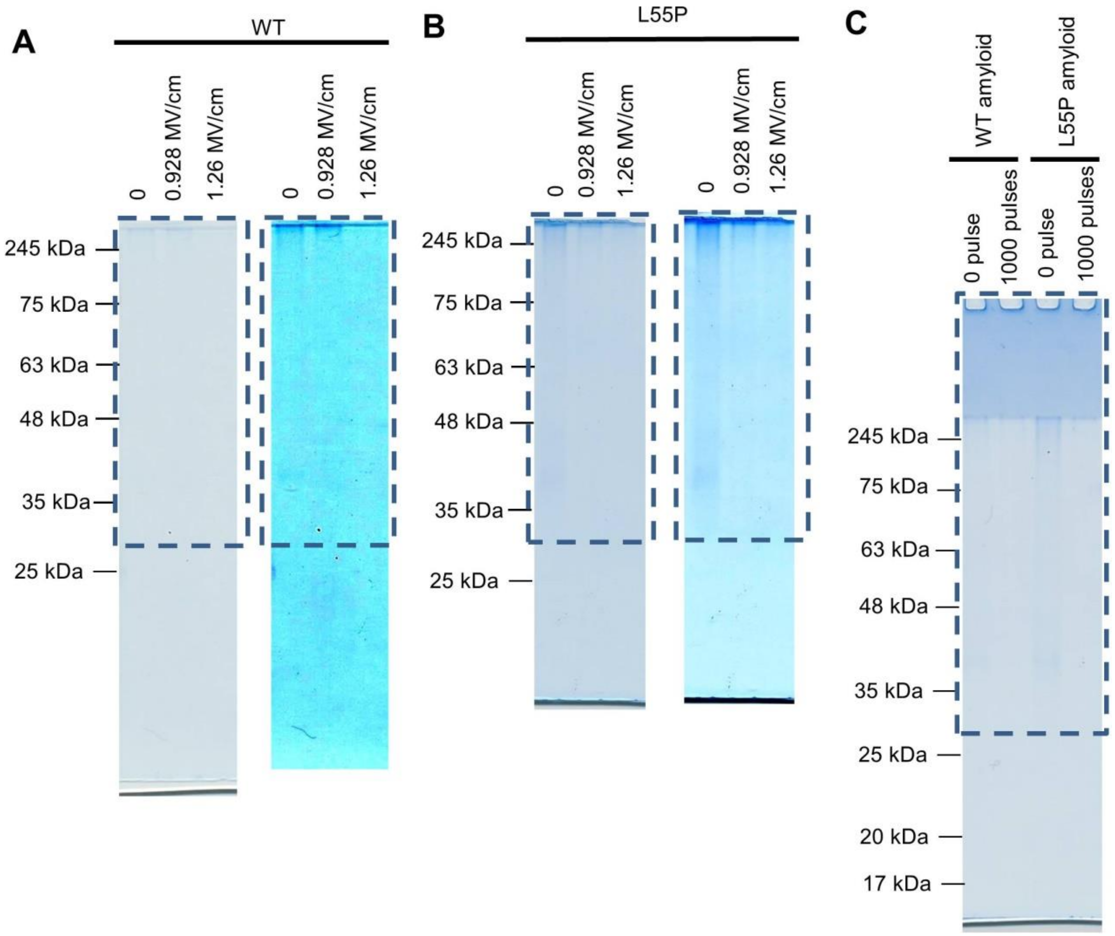

Supplementary Figure S4. Full photographs of gel electrophoresis in Fig. 4.

A and **B**: Full photos of Fig. 4A and B. Because the bands were too weak to check without contrast, contrast versions were produced. **C**: Full photograph without contrast of Fig. 4D. Image J adjusted Supplementary Figure S4A-C.

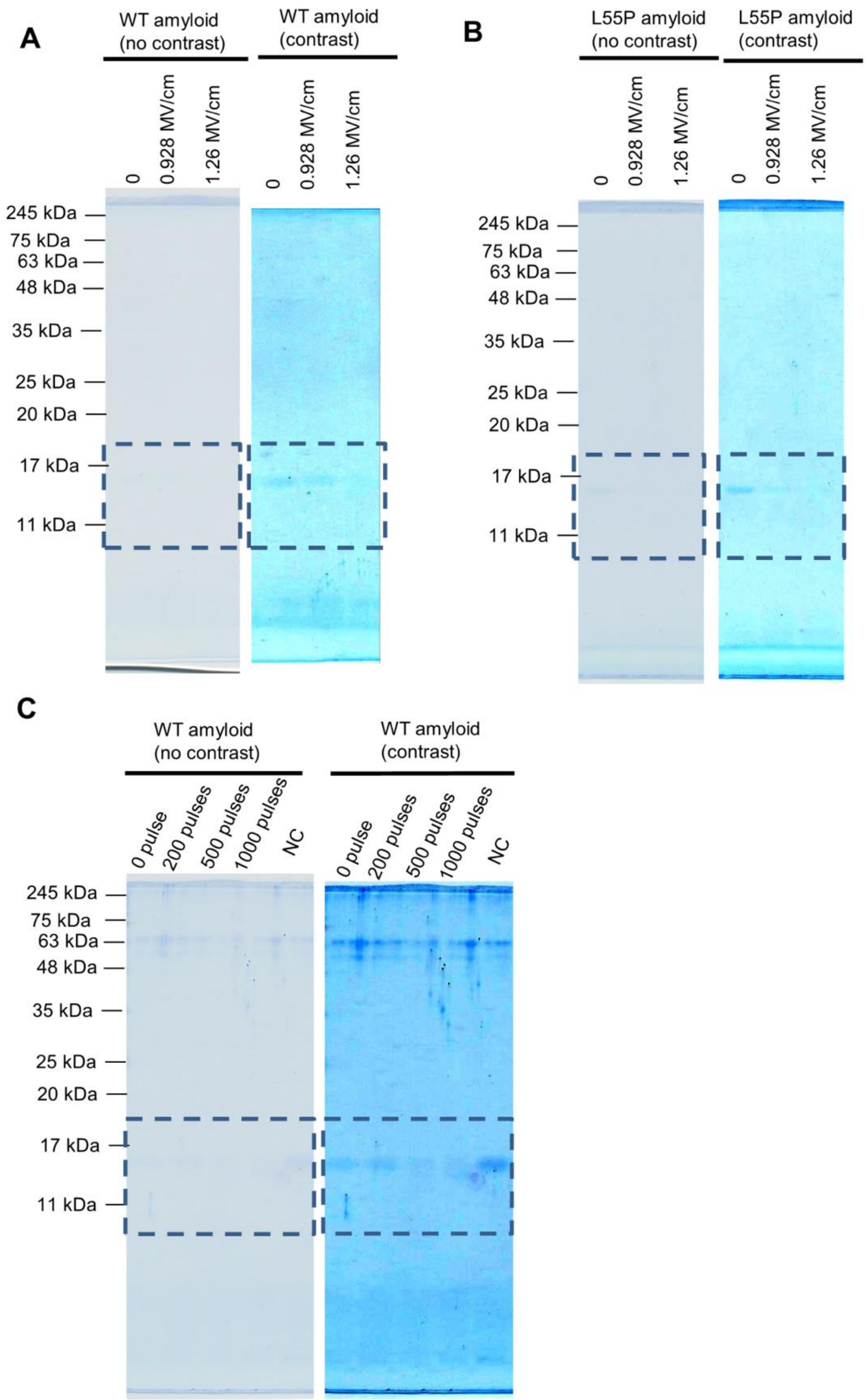
A
WT amyloid (no contrast)
WT amyloid (contrast)
0
0.928 MV/cm
1.26 MV/cm
245 kDa
75 kDa
63 kDa
48 kDa
35 kDa
25 kDa
20 kDa
17 kDa
11 kDa
B
L55P amyloid (no contrast)
L55P amyloid (contrast)
C
0 pulse
200 pulses
500 pulses
1000 pulses
NC

95

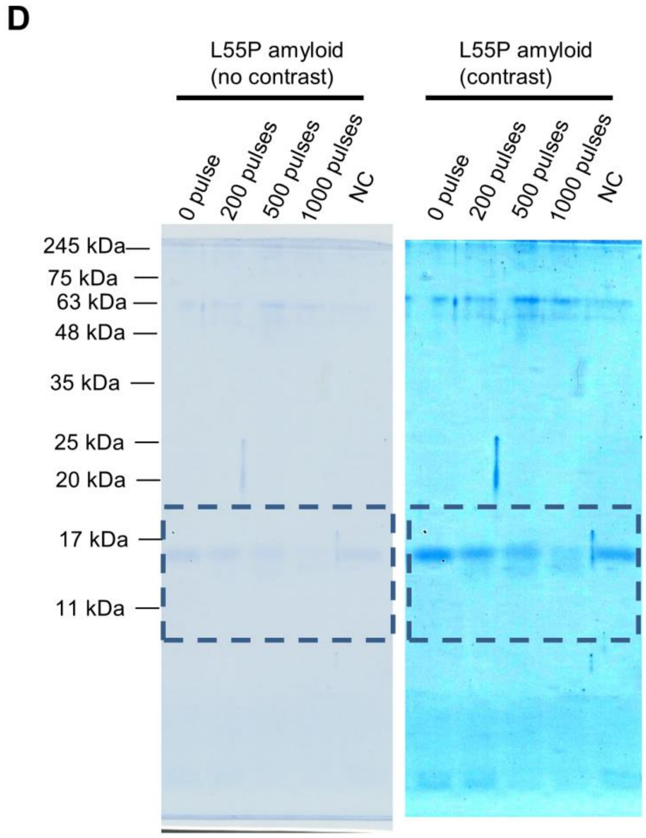

Supplementary Figure S5. Full photographs of gel electrophoresis in Fig. 5. Full sodium dodecyl sulfate-polyacrylamide gel electrophoresis (SDS-PAGE) photographs of WT (**A**) and L55P mutant (**B**) aggregate-derived subunits exposed to 1000 pulses at 0.928 and 1.26 MV/cm. Full SDS-PAGE photographs of WT (**C**) and L55P mutants (**D**). Aggregate-derived subunits exposed to several different pulses at 1.26 MV/cm. Image J adjusted Supplementary Figure S5A-D.

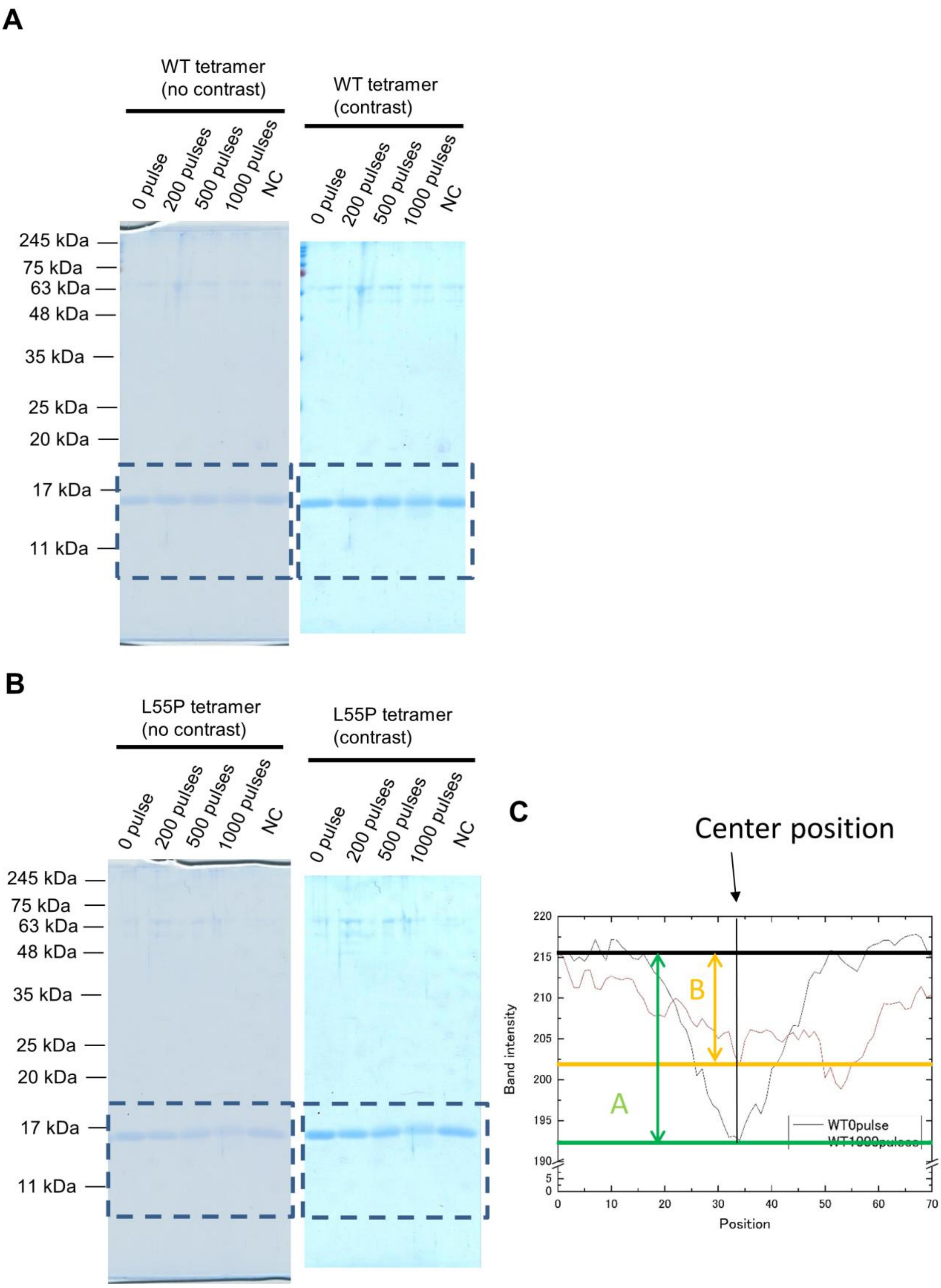

Supplementary Figure S6. Full photographs of gel electrophoresis in Fig. 6. Full SDS-PAGE photographs of WT (**A**) and L55P mutants (**B**). Tetramer-derived subunits

exposed to different pulses at 1.26 MV/cm. Because some bands were too weak to check without contrast, a contrast version was produced. **C**: The center-position-intensity ratios were calculated as B/A. A: Center-position intensity without pulse treatment, and B: center-position intensity with pulse treatment of 1.26 MV/cm at 1000 pulses. Image J adjusted Supplementary Figure S6A, and B.

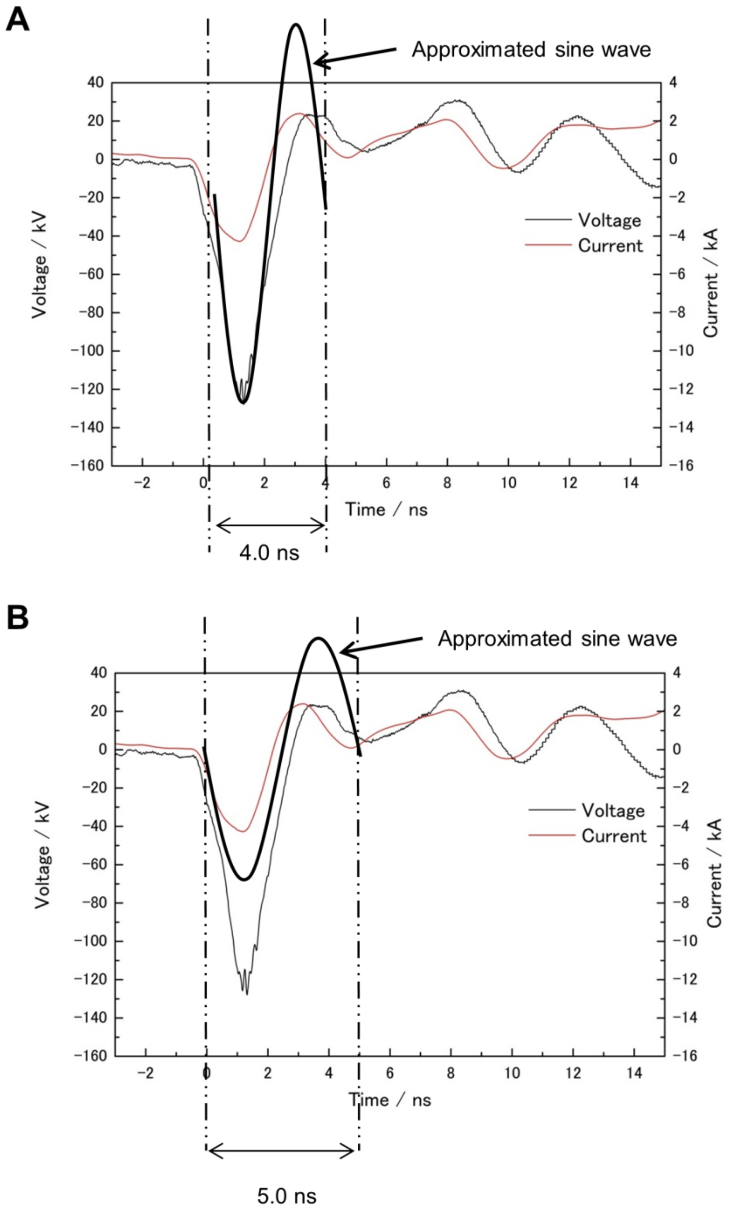

$$\omega = \frac{1}{4.0\ \mathrm{ns}} = 250\ \mathrm{MHz}$$

$$\omega = \frac{1}{5.0\ \mathrm{ns}} = 200\ \mathrm{MHz}$$

Supplementary Figure S7. Approximating the waveform near 126 kV by a sine wave. A: The sine wave used to approximate the upper part of the main voltage has a cycle length of 4.0 ns and a frequency of 250 MHz. **B**: A sine wave with a 5.0-ns cycle length and a frequency of 200 MHz approximates the lower part of the voltage wave.
